# A review of the incidence of tumor lysis syndrome in patients with chronic lymphocytic leukemia treated with venetoclax and debulking strategies

**DOI:** 10.1002/jha2.427

**Published:** 2022-04-05

**Authors:** Jeffrey P. Sharman, Juliana M. L. Biondo, Michelle Boyer, Kirsten Fischer, Michael Hallek, Dingfeng Jiang, Arnon P. Kater, Michele Porro Lurà, William G. Wierda

**Affiliations:** ^1^ Department of Medical Oncology Willamette Valley Cancer Institute and Research Center/US Oncology Research Eugene Oregon USA; ^2^ Genentech, Inc. South San Francisco California USA; ^3^ Roche Products Limited Welwyn Garden City UK; ^4^ University of Cologne, Faculty of Medicine and University Hospital Cologne, Department I of Internal Medicine, German CLL Study Group, Center for Integrated Oncology Aachen Bonn Cologne Dusseldorf Germany; ^5^ AbbVie North Chicago Illinois USA; ^6^ Department of Hematology Cancer Center Amsterdam Lymphoma and Myeloma Center Amsterdam Amsterdam University Medical Centers Amsterdam the Netherlands; ^7^ F. Hoffmann‐La Roche Ltd Basel Switzerland; ^8^ Department of Leukemia The University of Texas MD Anderson Cancer Center Houston Texas USA

**Keywords:** chronic lymphocytic leukemia, debulking, hospitalization, tumor lysis syndrome, venetoclax

## Abstract

We reviewed the literature (January 2010–June 2021) on the effectiveness of debulking strategies before venetoclax initiation in patients with chronic lymphocytic leukemia to reduce tumor burden, downgrade tumor lysis syndrome (TLS) risk, and avoid hospitalization. Low TLS incidence and reduced TLS risk based on tumor burden were reported following debulking in clinical trials. Real‐world observational studies reporting debulking regimens recorded no TLS events, and those without debulking strategies had greater TLS incidence. Debulking prior to venetoclax considerably reduces TLS incidence. Further clinical trials and real‐world studies may provide additional evidence on effectiveness of debulking in reducing TLS incidence and hospitalization need.

## 1 INTRODUCTION

Treatment outcomes for patients with chronic lymphocytic leukemia (CLL) have improved with the availability of targeted therapies, including the highly selective, potent, and orally bioavailable B‐cell lymphoma 2 inhibitor, venetoclax [1, 2, 3, 4]. Venetoclax has been shown to be effective and well‐tolerated for both the first‐line treatment of CLL and for relapsed/refractory (R/R) CLL [5, 6, 7, 8, 9]. Venetoclax potently induces CLL cell apoptosis and therefore is associated with risk for tumor lysis syndrome (TLS; a life‐threatening metabolic disorder [if not anticipated, monitored, and treated appropriately] that occurs when tumor cells undergo rapid disintegration, releasing their intracellular contents into the systemic circulation) during treatment initiation. Patients treated with venetoclax should be stratified by TLS risk and receive prophylactic measures, per the label guidance, as well as monitoring throughout treatment so that occurrences of TLS are identified early and can be managed (Table 1) [10, 11]. Information on the diagnosis and classification of TLS is provided in Supplementary Table S1. Development and adoption of a gradual venetoclax ramp‐up following initiation at a low dose of 20 mg daily, with risk‐based monitoring and interventions based on tumor burden, has mitigated events of clinical TLS and reduced the number of laboratory TLS events (Supplementary Tables S2 and S3). Further, “debulking” (to reduce as much of the volume/bulk of cancer cells as possible) with chemotherapy, immunochemotherapy, or targeted therapy treatment cycles prior to initiating venetoclax can potentially reduce tumor burden and, consequently, the need for hospitalization (by downgrading high‐risk tumor burden to medium‐ or low‐risk or medium‐risk with creatinine clearance [CrCl] < 80 mg/dl to low‐risk for TLS; Supplementary Table S2). Thus, debulking has helped to combat the severity and reduce the incidence of TLS [12, 13, 14, 15, 16, 17]. Despite these mitigating measures, there is still a risk of TLS, which may potentially increase in incidence as venetoclax becomes more widely used in real‐world settings [18].

**TABLE 1:** Summary of tumor lysis syndrome (TLS) prophylaxis and monitoring measures [48]

Prophylaxis measures			
Hydration	Patients should be instructed to drink plenty of water daily (1.5–2.0 L is recommended), starting 2 days before and throughout the dose‐titration phase, especially at each subsequent dose increase, to enable adequate hydration. Intravenous fluids should be administered for patients deemed at high risk of TLS or for those who cannot maintain an adequate level of oral hydration		
Anti‐hyperuricemic agents	Administered 2–3 days prior to venetoclax initiation in patients with high uric acid levels or at risk of TLS. May be continued through the titration phase		
Laboratory assessments	Pre‐dose: Blood chemistries should be assessed for all patients prior to the initial dose, to evaluate kidney function and correct pre‐existing abnormalities. Blood chemistries should be re‐assessed prior to each subsequent dose increase during the titration phase.		
	Post‐dose: For patients at risk of TLS, blood chemistries should be monitored 6–8 and 24 h after the first dose of venetoclax. Electrolyte abnormalities should be corrected promptly. Evaluation of the 24‐h blood chemistry results should occur before administration of the next dose of venetoclax. The same monitoring schedule should be followed at the start of the 50 mg dose and at subsequent dose increases for patients who continue to be at risk following reassessment		
Hospitalization	Based on physician assessment. Some patients, for example, those at high risk of TLS, may require hospitalization on the day of the first dose of venetoclax for more intensive prophylaxis and monitoring during the first 24 h. Hospitalization should be considered for subsequent dose increases based on a reassessment of risk		
Dose modifications for TLS	Venetoclax dose should be withheld the day after a patient experiences blood chemistry changes suggestive of TLS. If resolved within 24–48 h of the last dose, venetoclax can be resumed at the same dose. For events of clinical TLS or blood chemistry changes requiring more than 48 h to resolve, treatment should be resumed at a reduced dose, and this modified dose should be continued for 1 week before increasing the dose again		
	*Dose at interruption (mg)*	*Restart dose (mg)*	
	*400*	*300*	
	*300*	*200*	
	*200*	*100*	
	*100*	*50*	
	*50*	*20*	
	*20*	*10*	

The aim of the current review was to investigate the impact of debulking strategies prior to venetoclax initiation, especially their efficacy in reducing tumor burden and the need for hospitalization.

## 2 LITERATURE REVIEW

### 2.1 Search strategy

A comprehensive literature search of PubMed, Embase, and the Cochrane Central Register of Controlled Trials was undertaken from November 2020 to June 2021. The search used the free‐text terms “venetoclax,” “chronic lymphocytic leukemia,” “tumor lysis syndrome” and “debulking,” along with their other derivatives (ABT‐199, GDC‐0199, RG‐7601) and abbreviations (CLL, TLS).

### 2.2 Screening and selection of studies

Studies were eligible for inclusion if they were written in English and published between January 2010 and June 2021 in peer‐reviewed journals. Abstracts for presentations at scientific meetings (American Society for Clinical Oncology, American Society of Hematology, European Hematology Association, and International Conference on Malignant Lymphoma were also included.

After removing all duplicates, the initial search yielded 4475 results. Reviews, preclinical studies, case studies, and all articles that failed to mention both venetoclax and patients with CLL, as well as TLS and/or debulking, were then excluded. In total, 168 articles and abstracts were reviewed for relevance, of which 43 have been included in this review. Overall, 16 clinical trials (eight in patients with previously untreated CLL, three in patients with R/R CLL, and five reporting data for both groups) and 10 studies reporting real‐world data are covered by the 43 articles and abstracts.

## 3 DEBULKING FOR REDUCTION OF TLS RISK

### 3.1 Debulking strategies utilized and reported rates of TLS in clinical trials

In clinical trials, tumor debulking was most commonly achieved using obinutuzumab, ibrutinib, or bendamustine; however, other treatments were investigated, including umbralisib and ublituximab (U2), duvelisib, and acalabrutinib (Table 2). Notably, the timing of debulking varied significantly between clinical trials, occurring within the same cycle as venetoclax initiation in some studies and starting up to three cycles prior to venetoclax in others. No TLS‐related deaths were observed, and only low numbers of TLS events (very few of which were clinical) were reported following debulking regimens in clinical trials of venetoclax (Table 3).

**TABLE 2:** Summary of clinical trials included that used a debulking strategy prior to initiation of venetoclax

				Treatment			
Study	Trial number	Phase	Setting (TN, R/R, etc.)	Debulking regimen including duration	Venetoclax regimen	Overall duration	No. pts^a^
CLL14 [9]	NCT02242942	3	TN	G for 21 days (IV infusion 100 mg Day 1, Cycle 1 and 900 mg Day 2, Cycle 1 [or 1000 mg Day 1, Cycle 1]; 1000 mg Days 8 and 15, Cycle 1)	5‐week Ven ramp‐up started Day 22, Cycle 1 (20, 50, 100, 200, and 400 mg/day), then Ven (400 mg/day) until end of Cycle 12	Twelve cycles (VenG for six [28‐day] cycles, Ven monotherapy for six cycles)	212
HOVON 139/GIVe [19, 49]	The Netherlands Trial Registry ID no: #NTR604	2	TN	Two cycles of G monotherapy (Cycle 1, Day 1: 100 mg; Day 2: 900 mg; Day 8: 1000 mg; Day 15: 1000 mg; Cycle 2, Day 1: 1000 mg)	Ven weekly ramp‐up (20, 50, 100, 200, and 400 mg/day) started in Cycle 3, then followed with 400 mg/day	Two cycles of G, six cycles of VenG (Ven 400 mg daily, G Cycles 3–8 Day 1: 1000 mg) then Ven monotherapy for six cycles (Cycles 9–14: 400 mg/day). After that, pts in partial or full remission received maintenance with 12 cycles of Ven	65
GP28331 [20]	NCT01685892	1b	TN and R/R	G for 21 days (100 mg Day 1, 900 mg Day 2, 1000 mg Days 8 and 15)	Ven ramp‐up with weekly dose increases to target dose (cohort 1: 100 mg; cohort 2: 200 mg; cohort 3: 400 mg; cohort 4: 600 mg)	VenG for six (28‐day) cycles, followed by Ven monotherapy until PD, unacceptable toxicity, or death in R/R pts or 1‐year fixed treatment in TN pts	75
Rogers et al. Blood [21, 22]	NCT02427451	1b/2	TN and R/R	Two cycles of G (1000 mg IV given on Days 1–2 [split dose], 8 and 15 of Cycle 1 and Day 1 of Cycle 2). During Cycle 2 pts also received Ibr 420 mg/day orally	Ven started on Day 1, Cycle 3 at 20 mg orally daily, with dose ramp‐up every 7 days to 50, 100, 200, and 400 mg. Ven continued through Cycle 14	One cycle of G alone, one cycle of Ibr + G, six cycles of Ven + Ibr + G, then six cycles of Ibr + Ven. Ibr could be continued after Cycle 14 (investigator's discretion)	37
AVO [23]	NCT03580928	2	TN	One cycle with acalabrutinib (100 mg), then two cycles of acalabrutinib + G (100 mg Day 1, 900 mg Day 2, and then 1000 mg Days 8 and 15 of Cycle 1, and Day 1 of Cycles 2–6	Ven ramp‐up (from 20 mg to 400 mg) beginning at Cycle 4	One cycle of acalabrutinib, two cycles of acalabrutinib + G (AO) three cycles of triplet AO + Ven (AVO) therapy, then acalabrutinib + Ven (AV doublet) through Cycle 15	37
M16‐788/ CLL‐076 [24, 25]	NCT03406156	3b	TN	Two to six cycles of G or BG	5‐week Ven ramp‐up at the beginning of VenG for 5 months	Two to six cycles of debulking, VenG for 5 months, then Ven monotherapy for up to 53 weeks	110
CLL2‐BAG [26, 27]	NCT02401503	2	TN and R/R	Two cycles of B (70 mg/m^2^ IV on Days 1 and 2 for pts with ALC ≥25 × 10^9^ cells/L and lymph nodes > 5 cm in diameter), then one cycle of G (1000 mg IV on Days 1, 2, 8, and 15)	Weekly dose escalation of Ven over 5 weeks (20, 50, 100, 200, and 400 mg/day)	Two cycles of B (pts with ALC ≥25 × 10^9^ cells/L and lymph nodes > 5 cm in diameter), one cycle of G, five cycles of VenG, then up to 24 months of maintenance with VenG (Ven 400 mg daily with G 1000 mg every 12 weeks)	66
GO28440 [28]	NCT01671904	1b	TN and R/R	One cycle of B 90 mg/m^2^ (TN) or 70 mg/m^2^ (R/R) Days 1–2 and either R (375 mg/m^2^ Day 1) or G (100 mg Day 1, 900 mg Day 2, and 1000 mg Days 8 and 15)	Ven initiated using a weekly ramp‐up to target dose (cohort 1: 100 mg; cohort 2: 200 mg; cohort 3: 400 mg).	One cycle of BR followed by five cycles of Ven + BR or one cycle of BG, followed by five cycles of Ven + BG	82^b^ (Ven‐BR: 60; Ven‐BG: 22)
GLOW [50]	NCT03462719	3	TN	Three (28‐day) cycles of Ibr (420 mg/day)	5‐week ramp‐up of Ven (beginning at 20 mg to 400 mg) starting on Day 1, Cycle 4, then Ven 400 mg/day	Fifteen cycles (three cycles of Ibr monotherapy, then Ibr + Ven for 12 cycles) followed by Ibr alone until PD or unacceptable toxicity	106
CAPTIVATE [29, 30, 31, 32, 33, 34, 35]	NCT02910583	2	TN	Three (28‐day) cycles of Ibr (420 mg/day)	5‐week ramp‐up (Ven 20, 50, 100, 200, and 400 mg/day) starting on Day 1, Cycle 4, then Ven 400 mg/day	Ibr + Ven for 12 (28‐day) cycles, or until PD or unacceptable toxicity	164
Jain et al. N Engl J Med [36]	NCT02756897	2	TN, high‐risk and older pts	Three (28‐day) cycles of Ibr (420 mg/day)	Weekly Ven dose escalation to 400 mg/day starting on Day 1, Cycle 4	27 cycles (three cycles of Ibr monotherapy, then Ibr + Ven for 24 cycles) followed by Ibr alone until PD or unacceptable toxicity	75
Thompson et al. Blood [37, 38]	NCT03128879	2	Pts with high‐risk disease (TN or R/R)	Pts receiving Ibr therapy for ≥1 year	Weekly dose‐escalation of Ven (beginning at 20 mg/day, until 400 mg/day target reached)	Ibr + Ven could continue for up to 2 years	45
CLARITY [39, 40, 41, 42]	ISCRTN: 13751862	2	R/R	8 weeks of Ibr (420 mg/day)	Ven starting on Day 1, Week 9. Weekly dose ramp‐up from 10 mg/day for first three pts and 20 mg/day for all other pts (20, 50, 100, 200, and 400 mg/day)	26 months (Ibr monotherapy for 2 months, Ibr + Ven for 24 months)	54
BOVen [43, 44]	NCT03824483	2	TN	Two cycles of zanubrutinib (160 mg starting Day 1) plus G (1000 mg IV Day 1 [or split Days 1–2] Day 8 and 15 of Cycle 1, then Day 1 of Cycles 2–8)	Ven 5‐week ramp‐up initiated Cycle 3, Day 1 (beginning at 20 mg and increasing to 400 mg orally daily)		39
U2‐Ven [45]	NCT03379051	1/2	R/R	Three cycles of Umbra daily along with Ubli, administered weekly during Cycle 1, then once during Cycles 2 and 3	Ven ramp‐up to 400 mg in Cycle 4, given with Umbra for 9 cycles	Twelve cycles (three cycles for debulking, Umbra + Ven for nine cycles)	21
Crombie et al. Blood [46, 47]	NCT03534323	1	R/R	Duvelisib twice daily for 7 days	Ven started on Day 8 (at 10 mg/day, with weekly ramp‐up to 20, 50, 100, 200, and 400 mg; last three pts initiated Ven at 20 mg), given with duvelisib		22

Abbreviations: B, bendamustine; G, obinutuzumab; Ibr, ibrutinib; IV, intravenous; No., number; PD, disease progression; pts, patients; R, rituximab; R/R, relapsed/refractory; TN, treatment naïve, Ubli, ublituximab; Umbra, umbralisib; Ven, venetoclax.

^a^ Numbers represent only patients treated with venetoclax, patients treated in the control arm of randomized studies are not included within this count, neither are patients who did not receive study drug or did not initiate venetoclax;

^b^Includes patients treated with Schedule A (no debulking) and Schedule B (debulking).

**TABLE 3:** TLS rates and outcomes of TLS by debulking strategy, venetoclax regimen, and TLS risk

Clinical trial	Debulking regimen including duration	Venetoclax regimen	No. pts	TLS risk at baseline, n (%)	Total no. of pts with TLS (clinical events/ laboratory events)	Occurrence of TLS (during debulking or venetoclax treatment)	TLS treatment/management	TLS outcome (e.g., resolved, required hospitalization)
CLL14 [9]	G for 21 days (IV infusion 100 mg Day 1, Cycle 1 and 900 mg Day 2, Cycle 1 [or 1000 mg Day 1, Cycle 1]; 1000 mg Day 8 and 15 Cycle 1)	5‐week Ven ramp‐up started Day 22, Cycle 1 (20, 50, 100, 200, and 400 mg/day), then Ven (400 mg/day) until end of Cycle 12	212	Low: 29 (14) Medium: 139 (64) High: 48 (22)^b^	3^c^	NR	NR	NR
HOVON 139/GIVe [19, 49]	Two cycles of G monotherapy (Cycle 1 Day 1: 100 mg; Day 2: 900 mg; Day 8: 1000 mg; Day 15: 1000 mg; Cycle 2 Day 1: 1000 mg)	Ven weekly ramp‐up (20, 50, 100, 200, and 400 mg/day) started in Cycle 3, then followed with 400 mg/day	65	Medium: 41 (62) High: 19 (29)	6 (0/6)	Four during G debulking and two during Ven ramp‐up	Protocol‐mandated IV hydration and allopurinol rasburicase	All cases of TLS resolved
GP28331 [20]	G for 21 days (100 mg Day 1, 900 mg Day 2, 1000 mg Day 8 and 15)	Ven ramp‐up with weekly dose increases to target dose (cohort 1: 100 mg; cohort 2: 200 mg; cohort 3: 400 mg; cohort 4: 600 mg)	75	Low: 12 (16) Medium: 41 (55) High: 22 (29)	3 (0/4)	One laboratory TLS occurred after G but before Ven, the other three occurred during Ven ramp‐up	NR	NR
Rogers et al. Blood [21, 22]	Two cycles of G (1000 mg IV given on Day 1–2 [split dose], 8 and 15 of Cycle 1 and Day 1 of Cycle 2). During Cycle 2 pts also received Ibr 420 mg/day orally	Ven started on Day 1, Cycle 3 at 20 mg orally daily, with dose ramp‐up every 7 days to 50, 100, 200, and 400 mg. Ven continued through Cycle 14	37	For TN pts (*n* = 25) Medium: 18 (72) High: 7 (28)^d^ For R/R pts (n = 12) Low: 1 (8) Medium: 7 (58) High: 4 (33)	0	N/A	N/A	N/A
AVO [23]	One cycle with acalabrutinib (100 mg), then two cycles of acalabrutinib + G (100 mg Day 1, 900 mg Day 2, and then 1000 mg Day 8 and 15 of Cycle 1, and Day 1 of Cycles 2–6	Ven ramp‐up (from 20 mg to 400 mg) beginning at Cycle 4	37	NR	2 (0/2)	Both grade 3 laboratory TLS events occurred after G, but before Ven	NR	Both pts continued G
M16‐788/ CLL‐076 [24, 25]	Two to six cycles of G or BG	5‐week Ven ramp‐up at the beginning of VenG for 5 months	110	NR	10 (2/8)	Nine TLS events (including the two clinical TLS, five laboratory TLS, and two unclassified) during debulking, one laboratory TLS during VenG	NR	NR
CLL2‐BAG [26, 27]	Two cycles of B (70 mg/m^2^ IV on Days 1 and 2 for pts with ALC ≥25 × 10^9^ cells/L and lymph nodes > 5 cm in diameter), then one cycle of G (1000 mg IV on Days 1, 2, 8, and 15)	Weekly dose escalation of Ven over 5 weeks (20, 50, 100, 200, and 400 mg/day	66	Low: 7 (11) Medium: 39 (59) High: 18 (27)^e^	3^f^	One during B debulking, one in induction Cycle 1 with G, two in Cycle 3 and one in Cycle 4 with VenG	NR	NR
GO28440 [28]	One cycle of B 90 mg/m^2^ (TN) or 70 mg/m^2^ (R/R) Day 1–2 and either R (375 mg/m^2^ Day 1) or G (100 mg Day 1, 900 mg Day 2 and 1000 mg Day 8 and 15)	Ven initiated using a weekly ramp‐up to target dose (cohort 1: 100 mg; cohort 2: 200 mg; cohort 3: 400 mg).	82^g^	For pts in schedule A and B (without and with debulking, respectively) Low: 16 (19) Medium: 45 (54) High: 21 (25)	3 (1/2)	Both laboratory TLS events occurred prior to Ven. The clinical TLS event occurred on Day 29 (Day 1 of Cycle 2) after administration of Ven 50 mg	Standard‐of‐care measures	All TLS events resolved (both laboratory events did not lead to permanent discontinuation of any study drug)
GLOW [50]	Three (28‐day) cycles of Ibr (420 mg/day)	5‐week ramp‐up of Ven (beginning at 20 mg to 400 mg) starting on Day 1, Cycle 4, then Ven 400 mg/day	106	NR	NR	NR	NR	NR
CAPTIVATE [29, 30, 31, 32, 33, 34, 35]	Three (28‐day) cycles of Ibr (420 mg/day)	5‐week ramp‐up (Ven 20, 50, 100, 200, and 400 mg/day) starting on Day 1, Cycle 4, then Ven 400 mg/day	164	High: 39 (24)	1 (0/1)^a^	NR	NR	NR
Jain et al. N Engl J Med [36]	Three (28‐day) cycles of Ibr (420 mg/day)	Weekly Ven dose escalation to 400 mg/day starting on Day 1, Cycle 4	75	Low: 10 (15) Medium: 58 (72) High: 12 (13)^b^	3 (0/3)	NR	NR	NR
Thompson et al. Blood [37, 38]	Pts receiving Ibr therapy for ≥1 year	Weekly dose‐escalation of Ven (beginning at 20 mg/day, until 400 mg/day target reached)	45	NR	0	N/A	N/A	N/A
CLARITY [39, 40, 41, 42]	8 weeks of Ibr (420 mg/day)	Ven starting on Day 1, Week 9. Weekly dose ramp‐up from 10 mg/day for first three pts and 20 mg/day for all other pts (20, 50, 100, 200, and 400 mg/day)	54	NR	1 (0/1)	During 200 mg Ven dose	Ven interrupted for 7 days (until biochemical abnormalities resolved)	Pt subsequently ramped up to 400 mg/d of Ven with no additional TLS
BOVen [43, 44]	Two cycles of zanubrutinib (160 mg starting Day 1) plus G (1000 mg IV Day 1 [or split Day 1–2] Days 8 and 15 of Cycle 1, then Day 1 of Cycles 2–8)	Ven 5‐week ramp‐up initiated Cycle 3, Day 1 (beginning at 20 mg and increasing to 400 mg orally daily)	39	High: 17 (44)	0	N/A	N/A	N/A
U2‐Ven [45]	Three cycles of Umbra daily along with Ubli, administered weekly during Cycle 1, then once during Cycles 2 and 3	Ven ramp‐up to 400 mg in Cycle 4, given with Umbra for 9 cycles	21	Low: 7 (33) Medium: 12 (57) High: 2 (10)	0	N/A	N/A	N/A
Crombie et al. Blood [46, 47]	Duvelisib twice daily for 7 days	Ven started on Day 8 (at 10 mg/day, with weekly ramp‐up to 20, 50, 100, 200, and 400 mg; last 3 pts initiated Ven at 20 mg), given with duvelisib	22	NR	0	N/A	N/A	N/A

Abbreviations: AE, adverse event; B, bendamustine; G, obinutuzumab; Ibr, ibrutinib; IV, intravenous; N/A, not applicable; No., number; NR, not reported; pts, patients; R, rituximab; R/R, relapsed/refractory; TLS, tumor lysis syndrome; TN, treatment naïve, Ubli, ublituximab; Umbra, umbralisib; Ven, venetoclax.

^a^ Three laboratory AEs were reported but of these only one met Howard criteria.

^b^ Total number of patients with TLS risk at baseline is greater than the number of patients who received venetoclax treatment.

^c^ Number and type of TLS events are not specified.

^d^ TLS risk for TN patients only (*n* = 25).

^e^ Two patients were assessed after baseline.

^f^ One patient had one TLS event and two patients had two TLS events each, although the type of TLS event was not specified.

^g^ Includes patients treated with Schedule A (no debulking) and Schedule B (debulking). Sixty patients received Ven‐BR (both Schedule A and B) and 22 patients received Ven‐BG (both Schedule A and B).

Across studies of patients who received debulking with obinutuzumab prior to initiation of venetoclax, the aggregated number of clinical and/or laboratory TLS events was 18 in 502 patients (3.4%) [8, 9, 19, 20, 21, 22, 23, 24, 25]. Two events (11.1%) were clinical (both during debulking); 13 (72.2%) were laboratory (four during debulking, three following debulking but prior to venetoclax, five during venetoclax ramp‐up, and one during combination treatment); and three (16.7%) were unclassified (timing of occurrence is not reported). Similarly, both clinical and laboratory TLS (one clinical [during venetoclax ramp‐up]; 10 laboratory [nine during debulking and one during venetoclax treatment]; and two unclassified [both during debulking]) were reported for 13/182 (7.1%) patients who received debulking with bendamustine (monotherapy or combined with obinutuzumab or rituximab) prior to initiation of venetoclax [24, 25, 26, 27, 28].

Studies that included debulking with ibrutinib prior to initiation of venetoclax reported no cases of clinical TLS; laboratory TLS was reported for 5/338 (1.5%) patients (TLS definition was study‐dependent; therefore, Cairo–Bishop or Howard criteria were used, depending on the study, and timing of occurrence was only reported for one event [during venetoclax treatment]) [29, 30, 31, 32, 33, 34, 35, 36, 37, 38, 39, 40, 41, 42]. Alternative debulking regimens employing novel targeted agents (including U2, duvelisib, and zanubrutinib plus obinutuzumab) were also evaluated; these studies, which included a combined total of 82 patients, did not report any TLS events with venetoclax [43, 44, 45, 46, 47].

### 3.2 Effect of debulking on TLS risk reported in clinical trials

Several clinical trials have indicated a reduction in TLS risk following debulking, compared with baseline risk, highlighting the effectiveness of including a debulking regimen prior to initiation of venetoclax (Table 3).

#### 3.2.1 Obinutuzumab

In the Phase 3 CLL14 trial, patients were assessed by absolute lymphocyte count (ALC) for their TLS risk at baseline (prior to any study drug being administered). Overall, in the venetoclax–obinutuzumab arm, median ALC decreased from 55 × 10^9^ cells/L at baseline to 1.27 × 10^9^ cells/L (range: 0.2–83.7) on Cycle 1, Day 15 [48], indicating a reduction in TLS risk following obinutuzumab debulking (see the Appendix for further information on TLS risk from a later ad hoc analysis).

TLS risk reduced markedly in the Phase 2 Dutch‐Belgian Cooperative Trial Group for Hemato‐oncology (HOVON) 139/GIVe trial following debulking with two cycles of obinutuzumab monotherapy: 28% of patients were high risk, and 60% were medium risk prior to debulking; following debulking, only 1% of patients were high risk, and 15% were medium risk [49]. In the Phase 3b M16‐788/CLL‐076 trial, medium and high TLS risk (73% and 26% of patients, respectively) at baseline were mainly driven by increased ≥25 × 10^9^ cells/L in 85% of patients) [24]. The median decrease in lymph node size was 0.5 cm for obinutuzumab monotherapy [24]. After two cycles of debulking therapy, low TLS risk was achieved in 56/61 (92%) patients treated with Obinutuzumab [24]. This reduction in TLS risk may facilitate outpatient venetoclax initiation and thus reduce the need for hospitalization.

#### 3.2.2 Obinutuzumab–bendamustine

In the Phase 3b M16‐788/CLL‐076 trial, low TLS risk was achieved in 20/26 (77%) patients after two cycles of debulking with obinutuzumab–bendamustine. The median decrease in lymph node size was 3 cm for obinutuzumab–bendamustine from baseline to Cycle 2; this reduced further with more cycles of debulking [24].

#### 3.2.3 Obinutuzumab–Bruton's tyrosine kinase inhibitor

Debulking with two cycles of obinutuzumab–zanubrutinib reduced TLS risk in the Phase 2 BOVen trial; 15/17 (88%) patients classified as high risk on Cycle 1, Day 1, were downgraded to low/medium risk at venetoclax initiation [43]. Further, debulking with acalabrutinib–obinutuzumab in the Phase 2 AVO trial decreased the number of patients at medium‐to‐high risk: 31/32 (97%) at baseline, compared with 3/32 (9%) at venetoclax initiation [23]. Notably, reduction in TLS risk appeared greater when obinutuzumab was combined with a Bruton's tyrosine kinase inhibitor (zanubrutinib or acalabrutinib), compared with chemotherapy (bendamustine).

#### 3.2.4 Ibrutinib monotherapy

Debulking with ibrutinib monotherapy in the Phase 2 CAPTIVATE trial, the Jain et al. studies and the Phase 3 GLOW trial (NCT03462719) led to the downgrading of TLS risk: 94% of patients were downgraded from high risk at baseline to medium risk or low risk after three cycles of ibrutinib in CAPTIVATE (secondary endpoint) [29, 30, 31]; 80% of the high‐risk patients and 48% of the medium‐risk patients were downgraded in the Jain et al. study following three cycles of ibrutinib monotherapy [36]; and 85% of patients were downgraded from high risk at baseline to medium risk or low risk after three cycles of ibrutinib in GLOW [50]. Overall, 75% of patients in CAPTIVATE avoided hospitalization for venetoclax initiation due to downgrading of their TLS risk level following debulking [32].

Although several strategies are adopted for debulking (obinutuzumab monotherapy, obinutuzumab in combination with chemotherapy or a Bruton's tyrosine kinase inhibitor, and ibrutinib monotherapy), with debulking lasting different lengths of time depending on treatment (1–3 cycles), the inclusion of debulking within a clinical trial is beneficial. Reported numbers of TLS events were low, and hospitalization for TLS was rare due to the effectiveness of debulking regimens, adherence to study protocols, and compliance with the US Prescribing Information (USPI) [51] and European Summary of Product Characteristics (SmPC) [48] recommendations for venetoclax.

#### 3.2.5 Bendamustine monotherapy

Debulking with two cycles of bendamustine (for patients with an ≥25 × 10^9^ cells/L or lymph nodes with a diameter of > 5 cm, unless they had contraindications) in the Phase 2 CLL2‐BAG trial led to a reduction in TLS risk; ALC was normalized in 31 patients and reduced by ≥50% in 37 of 44 patients after bendamustine debulking (median ALC at baseline was 57.1 × 10^9^ cells/L, compared with 2.7 × 10^9^ cells/L after debulking) [26]. Median ALC was further reduced after the first induction cycle (0.9 × 10^9^ cells/L). TLS risk was reassessed by the treating physician for 14 patients and retrospectively by the German CLL Study Group (GCLLSG) for 21 patients. Downgrading of TLS risk category (before the start of venetoclax) from high risk to medium risk occurred for three patients by the treating physician and three by the GCLLSG and downgrading from medium risk to low risk occurred for seven patients reassessed by the treating physician and 18 by the GCLLSG.

### 3.3 Real‐world data on the use of debulking strategies and TLS risk with venetoclax

Although the evidence for the impact of debulking is of lower quality from real‐world studies, compared with data from clinical trials, there is some indication that previous therapy can affect TLS risk in some patients within these more heterogeneous populations. These retrospective descriptive papers are summarized here to further characterize how other investigators have evaluated this strategy.

No cases of clinical or laboratory TLS have been reported in real‐world studies that included debulking regimens, such as the retrospective Moffitt Cancer Center study (where patients at intermediate/high risk of developing TLS could receive debulking) [52]. Risk stratification for patients with R/R CLL at the Moffitt Cancer Center enabled 15/20 (75%) patients who were considered to be at medium or high risk of developing TLS to receive debulking therapy with rituximab, ofatumumab, or obinutuzumab prior to initiation of venetoclax [52]. Following debulking, TLS risk status was reduced to low risk in 12 patients based on reduction in ALC.

Real‐world studies in the R/R setting that did not report the use of a debulking strategy appeared to be associated with a greater incidence of TLS, compared with real‐world studies that used debulking, although direct comparisons across studies are not possible due to differences in study methodology. Two TLS events were reported for the UK CLL Forum (one case of clinical TLS defined by the Howard criteria, one separate case of biochemical TLS, and one case of isolated hyperphosphatemia) [53], while six patients (13%) developed laboratory TLS (three of whom also developed clinical TLS) in the Mayo clinic study [54]. Clinical TLS occurred in 2.7% of patients (*n* = 8), and laboratory TLS occurred in 5.7% of patients (*n* = 17) in the international collaboration of the CLL Collaborative Study of Real‐World Evidence (CORE) and UK CLL Forum [55]. Similarly, TLS occurred in 9.7% of patients (nine clinical events) in a retrospective cohort study of R/R CLL patients treated with venetoclax across 24 US and 42 UK academic and community centers in partnership with the UK CLL Forum and the CLL CORE [56]. It is likely that these similarities in outcomes are due to overlapping patient populations, as these different real‐world cohort studies (with the exception of the Mayo clinic study) all use centers from the UK CLL Forum and CORE [53, 55, 56].

Further, a UK multicenter study (170 patients from 11 centers; venetoclax monotherapy, *n* = 99 [58%]; venetoclax–rituximab, *n* = 68 [40%]; and venetoclax–obinutuzumab, *n* = 3 [2%]) reported seven TLS events (three clinical and four biochemical, of which six occurred in high‐risk patients); however, there was no indication if these centers were the same as those in the UK CLL Forum [57]. TLS was observed in 14 (22%) patients receiving venetoclax monotherapy in a French Innovative Leukemia Organization (FILO) study of the French compassionate use cohort [58], 10 (12%) patients in an observational study conducted in Austria, Germany, and Switzerland (after receiving venetoclax–rituximab) [59], and three (6.3%) patients in a Spanish observational study where venetoclax was initiated at least 9 months before inclusion in the study (venetoclax monotherapy [81%]; venetoclax–rituximab [4%]; venetoclax–obinutuzumab [2%]; venetoclax and other agents [13%]) [60]. A total of 17 (51.5%) patients developed TLS by laboratory criteria at venetoclax doses from 20 to 400 mg, five patients developed TLS by clinical criteria, ranging from grade 0 to 3 in severity (per Cairo–Bishop definition) in the Koenig et al. Ohio State University study [61]. However, Koenig et al. used a rapid venetoclax dose escalation, with dose increases every 1 or 2 days, if tolerated. In real‐world studies, information on laboratory monitoring and correct dose ramp‐up administration was not always available.

Data from the CLL CORE and UK CLL Forum study (real‐world studies without debulking prior to venetoclax initiation) indicate that planned hospitalizations for venetoclax administration are more frequent in patients with high TLS risk than low or medium TLS risk, in agreement with the label recommendations [55]. TLS risk classification by the investigator at the time of venetoclax initiation was high in 28%, medium in 32%, and low in 40% of patients, with at least one planned hospitalization during venetoclax dose escalation in 88%, 80%, and 56% of patients, respectively [55]. Furthermore, 65% of patients at high TLS risk had two or more planned hospitalizations during venetoclax dose ramp‐up. However, on the basis of available data (ALC and lymph node measurements), the TLS risk level was misclassified in 12.5% of patients, demonstrating a need for training and education on TLS risk determination [55].

In a real‐world study where most patients had genetically high‐risk CLL and were heavily pretreated (with a median of five prior lines of therapy), high tumor burden was associated with increased incidence of TLS with venetoclax treatment; 3/8 (38%) patients with low initial tumor burden prior to venetoclax without debulking developed laboratory TLS, compared with 11/20 (55%) and 3/5 (60%) patients with medium and high tumor burden, respectively [61]. The CLL CORE and UK CLL Forum cohort study of 297 patients indicated that there are other variables affecting the incidence of TLS in real‐world studies in the R/R setting. These include USPI‐defined CrCl ( <80 vs. ≥80 ml/min [51]; multivariate analysis: odds ratio [OR] 2.6; 95% confidence interval [CI]: 1.1–6.2; *p* = 0.031) and TLS risk group prior to initiation of venetoclax (multivariate analysis, high risk vs. low risk: OR 3.0; 95% CI: 1.7–5.4; *p* = 0.001) [55]. However, when investigating whether any specific prognostic factors serve as predictors for TLS risk in routine clinical practice, no association was observed between risk of TLS and age, sex, Rai stage, fluorescence in situ hybridization (specific analysis not defined), immunoglobulin heavy chain gene mutation status, serum β‐2‐microglobulin, lactate dehydrogenase, ALC, or largest lymph node size [54].

### 3.4 Authors’ perspectives

So far, as can be seen from the results described above, we conclude that it is the effectiveness of the debulking strategy rather than any specific treatment strategy that is important with regard to reducing the risk of TLS. Of note, obinutuzumab is currently the only treatment approved in combination with venetoclax for treatment‐naïve patients with CLL, with the recommendation to begin venetoclax ramp‐up following three weekly doses of obinutuzumab monotherapy. Of particular interest, we have noted that TLS occurring after debulking is mainly laboratory rather than clinical TLS, and no lethal event has occurred in studies using debulking strategies prior to applying venetoclax. Furthermore, few instances of TLS have been reported for either treatment‐naïve or R/R patients with CLL after a debulking strategy has been used, suggesting that line of therapy is not indicative of a higher risk of a TLS event.

The definitions of tumor burden based on lymph node size and leukocytosis, established early in venetoclax development, are still used today. Based on the vast amount of experience and well‐documented cases from clinical trials, the aim of future studies should be to re‐determine risk factors for TLS, with more reliance placed on radiology for review of scans, in particular for determination of the extent of adenopathy. Ongoing and upcoming clinical trials (Table 4) may help to provide further evidence on the utility of debulking in reducing TLS risk. Future clinical trials incorporating accelerated ramp‐ups (conducted in experienced academic centers with close inpatient monitoring) will provide further insights into patient subgroups with R/R CLL who could benefit from shorter ramp‐up regimens, despite the greater risk of TLS associated with venetoclax dose escalations shorter than 5 weeks. Additionally, further real‐world studies where debulking is incorporated into routine practice for patients who are deemed to be at high risk of TLS will help to show the effectiveness of debulking in reducing the incidence of TLS and the need for hospitalization. Education on correct monitoring procedures during venetoclax initiation and management of TLS events, as well as the use of debulking strategies and evaluation of TLS risk, can continue to help reduce TLS incidence in real‐world practice.

**TABLE 4:** Summary of ongoing and upcoming clinical trials for debulking prior to initiation of venetoclax

				Treatment		
Study	Trial number	Phase	Setting (TN, R/R, etc.)	Debulking regimen	Venetoclax regimen	Overall duration
FLAIR	ISRCTN01844152	3	TN	Ibr for 8 weeks	Ven dose ramp‐up for 5 weeks	Up to 6 years
ERADIC	NCT04010968	2	TN fit pts with medium‐risk CLL	Ibr (420 mg/day) for 3 months	Beginning at Month 4, a 5‐week Ven dose ramp‐up (20, 50, 100, 200, and 400 mg) then 400 mg/day continuously from Month 5 to the end of treatment, either Month 15 or Month 27	15 or 27 months of treatment (Ibr for 3 months, followed by Ven + Ibr)
Stanford	NCT03045328	2	R/R	Ibr daily for 8 weeks	Ven daily beginning on Week 9 Day 1	61 weeks (8 weeks Ibr, then 53 weeks Ibr + Ven) in the absence of PD or unacceptable toxicity
VISION	NCT03226301	2	R/R	Two cycles of Ibr (420 mg daily)	Beginning Cycle 3, pts receive Ven daily (ramp‐up at start)	Ibr + Ven for 15 cycles, then Ibr until PD/relapse
ALLIANCE A041702	NCT03737981	3	TN older pts	Two cycles of Ibr (daily) and G (IV over 4 hours on Day 1, 2, 8, and 15 of Cycle 1 and on Day 1 of Cycle 2)	Beginning Cycle 3, pts receive Ven daily	14 cycles in the absence of PD or unacceptable toxicity (Ibr + G for two cycles, Ibr + G + Ven for 12 cycles), then one cycle of Ibr. Following this Ibr PO QD every 28 days in the absence of PD or unacceptable toxicity
ML5046	NCT03701282	3	TN	Ibr daily and G IV over 4 h on Day 1, 2, 8, and 15 of Cycle 1 and on Day 1 of Cycle 2–6	Ven daily Cycle 3–14	19 cycles in the absence of PD or unacceptable toxicity
Columbia	NCT03609593	2	TN	Three cycles of B + R	5‐week dose ramp‐up to 400 mg daily. VenR for 12 cycles	15 months

Abbreviations: B, bendamustine; CLL, chronic lymphocytic leukemia; G, obinutuzumab; Ibr, ibrutinib; IV, intravenous; PD, disease progression; PO, orally; pts, patients; QD, once a day; R, rituximab; R/R, relapsed/refractory; TN, treatment naïve; Ven, venetoclax.

## 4 CONCLUSION

Debulking strategies have resulted in reduced tumor burden and shifts in classification from high to lower TLS risk prior to venetoclax initiation. This in turn has strongly decreased rates of clinical and lethal TLS events and also reduced the need for hospitalization for venetoclax dosing in clinical studies. The importance to adhere to the 5‐week ramp‐up monitoring was recently reemphasized with the inclusion of more detailed guidance in the SmPC aligning to the USPI. This suggests that although mitigation of TLS risk with venetoclax is achieved by adherence to the established venetoclax initiation protocol (five weekly dose ramp‐up schedule, TLS prophylaxis, and monitoring measures), some improvement in aligning outpatient procedures with the measures recommended in the label is required. Nevertheless, debulking prior to the initiation of venetoclax may provide the opportunity for carefully selected patients to start venetoclax therapy in the outpatient setting.

## AUTHOR CONTRIBUTION

All authors were involved in the conception and design of the literature search, analysis and interpretation of the included publications, and the writing, review, and revision of the manuscript.

## CONFLICT OF INTEREST

Jeffrey P. Sharman reports stock options with Centessa Pharmaceuticals; consultancy with AbbVie, AstraZeneca, Beigene, Bristol Myers Squibb, Lilly, Pharmacyclics, and TG Therapeutics; and membership on board of directors or advisory committees for Centessa Pharmaceuticals. Juliana M.L. Biondo is an employee of Genentech, Inc. and has stock ownership for F. Hoffmann‐La Roche Ltd. Michelle Boyer is an employee of F. Hoffmann‐La Roche Ltd. Kirsten Fischer received honoraria and held a consulting/advisory role from F. Hoffmann‐La Roche Ltd and AbbVie; and received travel grants, accommodations and expenses from F. Hoffmann‐La Roche Ltd. Michael Hallek received grants, non‐financial support and personal fees (honoraria, speaker's bureau and/or advisory board) from AbbVie, Celgene, F. Hoffmann‐La Roche Ltd, Gilead, Mundipharma, Janssen, and Pharmacyclics. Dingfeng Jiang is an employee of AbbVie; and may own AbbVie stock or stock options. Arnon P. Kater has held a consultant or advisory role for Biogeneration and LAVA; received honoraria from Celgene, F. Hoffmann‐La Roche Ltd./Genentech, Inc., AstraZeneca, and Janssen; and received research funding from Celgene, F. Hoffmann‐La Roche Ltd./Genentech, Inc., AstraZeneca, and Janssen. Michele Porro Lurà is an employee of F. Hoffmann‐La Roche Ltd; and has stock ownership for F. Hoffmann‐La Roche Ltd. William G. Wierda is employed by The University of Texas MD Anderson Cancer Center; and received research funding from AbbVie and Genentech, Inc; received travel accommodations and expenses from AbbVie and Genentech, Inc.

## ETHICS STATEMENT

No experiments were performed on humans or animals, only previously published data were included.

## Supporting information

Supporting Information

## Data Availability

Data sharing is not applicable to this article as no new data were created or analyzed in this study. All information discussed within the literature review is derived from articles and abstracts published between January 2010 and June 2021. As such, the data are all included within the manuscript and referenced accordingly.
