# Supplementary material for: A review of the incidence of tumor lysis syndrome in patients with chronic lymphocytic leukemia treated with venetoclax and debulking strategies
Source: EJHaem. 2022 Apr 5;3(2):492–506. doi: 10.1002/jha2.427 (PMC9175963; doi:10.1002/jha2.427)
Supplement: Supplementary file 1 — Supporting Information [file JHA2-3-492-s001.docx]

**APPENDIX: SUPPLEMENTAL DATA**

Sharman et al. **A review of the incidence of tumor lysis syndrome in patients with CLL treated with venetoclax and debulking strategies**

**Effect of debulking with obinutuzumab on TLS risk reported in clinical trials**

An ad-hoc analysis of TLS risk following 3 weeks of obinutuzumab debulking in patients treated with venetoclax–obinutuzumab in CLL14 found that ALC reduced to normal (from ≥25 × 10^9^ cells/L to <25 × 10^9^ cells/L) after one cycle of obinutuzumab in 34 of 37 (92%) high-risk patients with lymph nodes <10 cm (by radiologic assessment) at baseline (missing ALC for two patients [5%]; data on file). This indicates that the TLS precaution measures could have been lowered for many patients, who would have fallen into a lower risk category if reassessment of TLS risk after obinutuzumab debulking was done per protocol, rather than retrospectively. Thus, the CLL14 study demonstrates the potential to reduce hospitalization needs after one cycle of obinutuzumab monotherapy.

**REFERENCES:**

1. Cairo MS, Bishop M. Tumour lysis syndrome: new therapeutic strategies and classification. British journal of haematology 2004;127(1):3-11.
2. Howard SC, Jones DP, Pui CH. The tumor lysis syndrome. The New England journal of medicine 2011;364(19):1844-54.
3. VENCLEXTA (Venetoclax) [package insert]. Abbvie Inc. Available at: <https://www.accessdata.fda.gov/drugsatfda_docs/label/2019/208573s013lbl.pdf>.
4. Venclyxto: Summary of product characteristics. AbbVie Inc. Available at: <https://www.ema.europa.eu/en/documents/product-information/venclyxto-epar-product-information_en.pdf>.
5. Jones GL, Will A, Jackson GH, Webb NJ, Rule S. Guidelines for the management of tumour lysis syndrome in adults and children with haematological malignancies on behalf of the British Committee for Standards in Haematology. British journal of haematology 2015;169(5):661-71.
6. Roberts AW, Davids MS, Pagel JM, et al. Targeting BCL2 with Venetoclax in Relapsed Chronic Lymphocytic Leukemia. The New England journal of medicine 2016;374(4):311-22.
7. Fischer K, Al-Sawaf O, Bahlo J, et al. Venetoclax and Obinutuzumab in Patients with CLL and Coexisting Conditions. The New England journal of medicine 2019;380(23):2225-36.
8. Al-Sawaf O, Zhang C, Tandon M, et al. Venetoclax plus obinutuzumab versus chlorambucil plus obinutuzumab for previously untreated chronic lymphocytic leukaemia (CLL14): follow-up results from a multicentre, open-label, randomised, phase 3 trial. The Lancet Oncology 2020;21(9):1188-1200.
9. Seymour JF, Kipps TJ, Eichhorst B, et al. Venetoclax-Rituximab in Relapsed or Refractory Chronic Lymphocytic Leukemia. The New England journal of medicine 2018;378(12):1107-20.
10. Seymour JF, Davids MS, Roberts AW, et al. Mitigation of tumor lysis syndrome (TLS) complications with venetoclax (VEN) in CLL. HemaSphere 2018;2(S1):848.
11. Davids MS, Hallek M, Wierda W, et al. Comprehensive Safety Analysis of Venetoclax Monotherapy for Patients with Relapsed/Refractory Chronic Lymphocytic Leukemia. Clinical cancer research : an official journal of the American Association for Cancer Research 2018;24(18):4371-9.
12. Seymour JF, Gribben JG, Davids MS, et al. Assessment of Tumor Lysis Syndrome in Patients with Chronic Lymphocytic Leukemia Treated with Venetoclax in the Clinical Trial and Post-Marketing Settings. Blood 2020;136(Suppl 1):37-8.
13. Koenig KL, Huang Y, Dotson EK, et al. Safety of venetoclax rapid dose escalation in CLL patients previously treated with B-cell receptor signaling antagonists. Blood advances 2020;4(19):4860-3.
14. Davids MS, Shadman M, Parikh SA, et al. A Multicenter, Retrospective Study of Accelerated Venetoclax Ramp-up in Patients with Relapsed/Refractory Chronic Lymphocytic Leukemia. Blood 2020;136(Suppl 1):51-2.

**Supplemental Table 1.** TLS diagnosis criteria and classifications

|  | **Cairo–Bishop criteria:^1^** | **Howard criteria:^2^** |
| --- | --- | --- |
| **Laboratory TLS event:**  clinically silent TLS, only detected through laboratory work-up | Occurring within 3 days before, or 7 days after, treatment initiation:   - Absolute values: ≥2 defined laboratory abnormalities - Relative changes: ≥25% decrease from baseline in serum calcium, and/or ≥25% increase from baseline in the serum values of uric acid, potassium, or phosphorous | Occurring within a 24-hour period:   - Absolute values:  ≥2 defined laboratory abnormalities |
| **Clinical TLS event:**  laboratory TLS accompanied by increased creatinine level, or when clinical manifestations are observed | Laboratory TLS plus ≥1 clinical manifestation, such as acute kidney injury with an elevated serum creatinine ≥1.5 times the upper limit of normal, cardiac arrhythmias, seizures or death | Laboratory TLS plus acute kidney injury, symptomatic hypocalcemia, or dysrhythmia |

TLS, tumor lysis syndrome

**Supplemental Table 2.** Risk factors for TLS and approaches used to mitigate risk

| **Risk factor** | **Mitigation** |
| --- | --- |
| Tumor burden (low-risk):  All LNs <5 cm AND ALC <25 x 10^9^ cells/L^3^  Tumor burden (medium-risk): Any LN 5 to <10 cm OR an ALC ≥25 x 10^9^ cells/L^3^ | Low/medium-risk: receive venetoclax dose escalation in an outpatient setting (with hospitalization considered for patients who are at medium risk with CrCl <80 mL/min)^3^ |
| Tumor burden (high-risk):  Any LN ≥10 cm OR ALC ≥25 x 10^9^ cells/L AND any LN ≥5 cm^3^ | High-risk: typically require hospitalization for venetoclax initiation, first dose escalation and potentially for further dose escalations^3^ |
| Reduced renal function (CrCl <80 mL/min)^4^ | More intensive approach to monitoring and prophylaxis for TLS at treatment initiation and dose titration^4^  No dose adjustment needed for patients with mild, moderate or severe renal impairment (CrCl ≥15 mL/min and <90 mL/min), but venetoclax pharmacokinetics have not been determined for patients with CrCl <15 mL/min or those on dialysis^4^ |
| Concomitant use of drugs that increase uric acid levels^5^ | Identify patients at the highest risk of TLS who could benefit from treatment with urolytic agents or tumor reduction^5^ |
| Increased age^5^ | – |
| Treatment with highly active agents^5^ | – |

ALC, absolute lymphocyte count; CrCl, creatinine clearance; LN, lymph node

**Supplemental Table 3.** Venetoclax dose-escalation regimens

|  | **Venetoclax dose** | **Ramp-up schedule** | **Incidence of TLS^a^** |
| --- | --- | --- | --- |
| **Phase 1 dose-finding study**^6^ | 200 mg or 100 mg for the first patient cohort, then 50 mg or 20 mg for subsequent patients | 3 weeks from 50 mg to target group dose, including step-up dose in week 2 | 18% (10/56; 3 clinical, 7 laboratory)^b^ |
| **Risk-based initiation**^7–12^ | Starting dose of 20 mg | 5-week ramp-up | CLL14: 1.4% (3/212)^7,8b^ MURANO: 3.1% (6/194)^9b^ M13-982/M14-032: 2.4% (4/168) ^10b^ M12-175/ M13-982/M14-032: 3.0% (5/166)^11b^ Pooled analysis: 1.8% (20/1138; 5 clinical, 15 laboratory)^12b^ |
| **Rapid dose-escalation**^13,14^ | 20 mg to  400 mg^13^ | Dose increases every 1–2 days if no TLS occurred^13^ | 66.7% (22/33; 5 clinical, 17 laboratory)^13c^ 19.4% (7/36; 2 clinical, 5 laboratory)^14d^ |

^a^No. of patients with TLS event; ^b^Identified using the Howard criteria^2^; ^c^Identified using the Cairo-Bishop criteria^1; d^Data are from a retrospective chart review from four large academic US medical centres

TLS, tumor lysis syndrome
